# Retinitis pigmentosa elicits neurodegeneration within the visual pathway in *REEP6* knockout mice

**DOI:** 10.4103/NRR.NRR-D-25-00168

**Published:** 2025-09-29

**Authors:** Yin Yang, Maoxia Lv, Binbin Qiao, Zhengjiang Yang, Houbin Zhang, Zhengzheng Wu, Yang Xia, Dezhong Yao, Ke Chen

**Affiliations:** 1 Department of Ophthalmology, Sichuan Provincial People’s Hospital, School of Medicine, University of Electronic Science and Technology of China, Chengdu, Sichuan Province, China; 2 Sichuan Provincial Key Laboratory for Human Disease Gene Study and the Center for Medical Genetics, Department of Laboratory Medicine, Sichuan Academy of Medical Sciences and Sichuan Provincial People’s Hospital, University of Electronic Science and Technology of China, Chengdu, Sichuan Province, China; 3 Research Unit for Blindness Prevention, Chinese Academy of Medical Sciences (2019RU026), Sichuan Academy of Medical Sciences and Sichuan Provincial People’s Hospital, Chengdu, Sichuan Province, China; 4 Department of Ophthalmology, Chongzhou People’s Hospital, Chengdu, Sichuan Province, China

**Keywords:** microglial activation, nerve regeneration, neurodegeneration, phosphorylated Tau, photoreceptor degeneration, primary visual cortex (V1), *REEP6*, retinitis pigmentosa, rod photoreceptors, visual pathway

## Abstract

While degenerative diseases of the central nervous system are commonly linked to age-related macular degeneration and glaucoma, they have also been infrequently associated with retinitis pigmentosa, a condition defined by retinal degeneration that can be caused by an isoform of receptor expression enhancing protein 6 (*REEP6*) expressed in rod photoreceptors. In this study, we used *REEP6* knockout mice (*REEP6*^–/–^) and wild-type mice (*REEP6*^+/+^) to examine neurodegenerative pathology within the visual pathways and neural activity in the primary visual cortex (V1) at three specific time points (1, 6, and 10 months) during retinitis pigmentosa progression. Microglial activation was observed in both the retina and the primary visual cortex starting at 1 month of age, but no such activation was detected in the lateral geniculate nucleus at any time point. Not only was increased microglial activation observed at 6 and 10 months within the primary visual cortex of *REEP6*^–/–^ mice, but also coinciding with elevated levels of phosphorylated Tau expression. At 6 and 10 months of age, primary visual cortex neurons in *REEP6*^–/–^ mice exhibited reduced responses to grating stimuli and increased spontaneous activity compared with neurons in the primary visual cortex of mice in the control group. Our findings show that retinitis pigmentosa induces neurodegenerative pathology within the visual pathway of mice, particularly in the primary visual cortex, suggesting that ocular disease contributes substantially to central nervous system degeneration. It may provide new clues for the selection of treatment opportunities and the development of therapeutic measures for the subsequent treatment of retinitis pigmentosa or even other retinal degenerative diseases.

## Graphical abstract

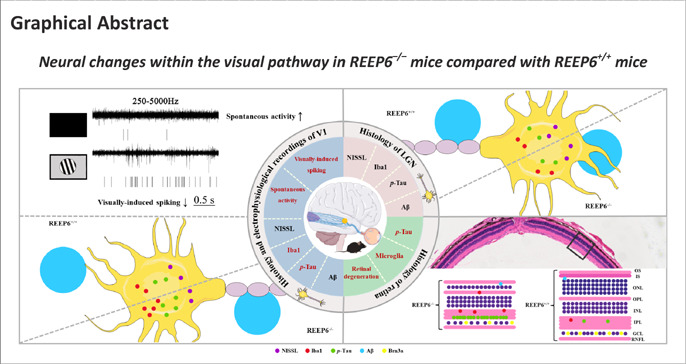

## Introduction

The substantial connection between the eyes and the central nervous system (CNS) is evidenced by ocular changes observed in various neurodegenerative CNS disorders, where visual manifestations occasionally precede major symptoms (Snyder et al., 2021). Additionally, several retinal degenerative diseases are associated with alterations in brain structure (Yan et al., 2017; You et al., 2021; Yang et al., 2024). Moreover, a range of etiological factors contribute to the neurodegeneration and cell loss seen in both retinal and CNS disorders, including oxidative stress, neuroinflammation, proteolytic degradation, hemodynamic dysregulation, trans-synaptic degenerative changes, genetic factors, and aberrant cellular signaling (Wang and Mao, 2021). The primary cause of permanent blindness in several ocular diseases, such as glaucoma, age-related macular degeneration (AMD), diabetic retinopathy (DR), and retinitis pigmentosa (RP), is degeneration of specific retinal neurons (Ahmad et al., 2020; Marchesi et al., 2021). RP constitutes a group of the most common and severe hereditary retinal degenerative diseases, which ultimately result in bilateral blindness (Karuntu et al., 2025). RP is characterized by progressive deterioration of rods during the initial phase, followed by subsequent degeneration of cones as the disease advances (Gregory-Evans et al., 2012). RP demonstrates significant genetic and phenotypic heterogeneity; indeed, mutations in over 90 genes have been associated with RP. These genes include those exhibiting autosomal dominant inheritance patterns (31 causative genes), autosomal recessive inheritance patterns (66 causative genes), and X-linked inheritance patterns (three causative genes), while unknown inheritance patterns account for approximately 40% to 50% of cases. Rarely, mitochondrial genes or digenic mutations have been implicated in RP development (Zeviani and Carelli, 2021). Recent studies have shown that mutations in the receptor expression enhancing protein 6 (*REEP6*) gene, which encodes receptor expression enhancing protein 6, can cause autosomal recessive RP (Lin et al., 2020; Zhang et al., 2021). REEP6 is a critical factor shaping the endoplasmic reticulum (ER), as it plays an essential role in facilitating cargo transport via a specific subset of clathrin-coated vesicles, enabling their movement from the ER to membrane locations containing Syntaxin3 within retinal rod photoreceptors (Veleri et al., 2017). REEP6 deficiency results in the dysfunction and eventual demise of rod cells (Lin et al., 2020). In the *REEP6*^–/–^ mouse model, photoreceptor degeneration is evident at as early as 2 months of age. By 3 months, the outer nuclear layer (ONL) in the *REEP6*^–/–^ retina contains approximately 8 nuclei, compared with the 11 to 12 nuclei present in wild-type controls. Furthermore, by 6 months of age, the outer segments of photoreceptors in the *REEP6*^–/–^ retina are significantly shortened and nearly undetectable. At 12 months, only one or two layers of photoreceptor nuclei with truncated inner segments remain in the *REEP6*^–/–^ retina. Complete degeneration of photoreceptors occurs by 18 months (Veleri et al., 2017). Most variants identified in humans are predicted to be loss-of-function mutations leading to complete absence of protein expression. Additionally, control animals with intact REEP6 exhibit normal dark-adapted electroretinography responses and preserved retinal architecture.

Neural circuits undergo substantial reorganization as a result of retinal degeneration, which in turn affects the activation of various neuronal populations responsible for encoding visual inputs and transmitting them to the visual system. Consequently, the altered neural signals transmitted via the optic nerve from a degenerated retina can induce subsequent rewiring of higher-order visual centers in the brain, thereby modifying visual information processing (Caravaca-Rodriguez et al., 2022). Prior studies have documented visually-evoked neural activity in the primary visual cortex (V1) of rodent models of retinal degeneration (S334ter-3 and RCS rats) and found that it was characterized by increased spontaneous activity, diminished stimulus selectivity, and impaired cortical processing of pattern stimulation across a broad range of spatial frequencies (Wang et al., 2018; Chen et al., 2020). Furthermore, recordings at the level of individual neurons, neuronal networks, or entire brain regions reveal heightened excitability and compromised visual processing in the early stages of Alzheimer’s disease (AD). As such, degeneration resulting from both ocular and cerebral disorders manifests as abnormal neural activity, disrupted information processing, and other distinctive features within the higher visual cortex (Wang et al., 2016). Cohort studies have demonstrated a robust correlation between retinal degeneration and brain degeneration (Shi et al., 2025); however, the existing evidence is insufficient to conclusively establish causality. To further investigate this issue, we examined morphological features and immunohistochemical markers associated with neurodegeneration within the visual pathways, including the retina, lateral geniculate nucleus (LGN), and V1, as well as neural activity within V1 during RP progression, in RP mice (*REEP6*^–/–^) and wild-type mice (*REEP6*^+/+^). This experiment has extended the study of the mechanism of RP disease to the central nervous system, hoping to provide a new perspective for the selection of treatment timing and the development of treatment measures in the future, promoting the development of human eye health.

## Methods

### Animals

REEP6 knockout mice (*REEP6*^–/–^, a model of retinal degenerative disease, were generated from C57BL/6J mice using CRISPR technology. The parental C57BL/6J (Cat# VSM10001, RRID: IMSR_JAX:000664) background mice were obtained from Beijing Viewsolid Biotechnology Co. Ltd. (Beijing, China; Certification No. SYXK (jing) 2025-0006) and maintained under specific-pathogen-free (SPF) conditions. *REEP6*^–/–^ mice were subsequently backcrossed to the C57BL/6J genetic background. Genetic characterization of the mice was conducted at 20 days postnatally. The knockout mouse is a global knockout line, which means that the gene is deleted in every cell, and no cell expresses REEP6 in these mice. Fifteen male *REEP6*^–/–^ mice were used in this study, including three 1-month-old (P1m) mice, six 6-month-old (P6m) mice, and six 10-month-old (P10m) mice, as well as fifteen age- and sex-matched *REEP6*^+/+^ control mice (REEP6 wild-type). All animals used in this experiment were drug-naïve and housed in separate, ventilated cages at a temperature of 22–25°C and a relative humidity of 50%–60%, with a 12/12-hour light/dark cycle and *ad libitum* access to food and water, at the animal facility of Sichuan Provincial People’s Hospital. All mice were treated in accordance with the ARVO Statement for the Use of Animals in Ophthalmic and Vision Research. All of the experimental procedures were approved by the Animal Management Committee of the University of Electronic Science and Technology of China (approval No. 1061423030125881) on May 20, 2023 and are reported according to Animal Research: Reporting of *In Vivo* Experiments (ARRIVE) guidelines (Percie du Sert et al., 2020).

### Histology

Once the electrophysiological recordings had been obtained, the mice were euthanized in accordance with humane guidelines with gaseous anesthetic (isoflurane, 4%–5%, RWD Life Science, Shenzhen, China). Subsequently, their brains and eyes, which had been pre-marked with nasolateral indicators, were carefully removed. The excised brain tissues were immediately placed in a 10% neutral buffered formalin solution (PH0996, Phygene Company, Fuzhou, China) for 48 hours, whereas the eyeballs were fixed in Davidson’s Fixative (PH0975, Phygene Company). Following fixation, both tissues were dehydrated in graded ethanol solutions and incubated in 100% xylene (A27, Kelong, Chengdu, China). Then, the samples were embedded in paraffin wax and sectioned using standard histological methods (Slicing Machine HM 340E, Thermo Fisher Scientific, Waltham, MA, USA). Specifically, whole paraffin-embedded mouse brains were sliced with a microtome into 4-μm-thick sections, focusing on bregma –2.92 mm. The coronal plane position was adjusted based on hippocampal size to ensure standardization of hippocampal dimensions while maintaining uniformity in brain section location. Retinal sections were obtained by slicing the eyes vertically through the pupil–optic nerve plane.

For immunofluorescence staining, the tissue samples were first deparaffinized with xylene and subsequently rehydrated in a graded ethanol series (100%, 95%, 85%, and 75% ethanol; 5 minutes per solution). To improve antibody accessibility, the sections were immersed in citrate antigen retrieval solution (pH 6.0; MVS-0101; Fuzhou Maixin Biotech, Ltd., Fuzhou, China) and microwaved to ensure that they reached room temperature, then rinsed with phosphate-buffered saline (PBS). To minimize non-specific labeling caused by autofluorescence, the specimens were treated with an autofluorescence quencher (G1221, Servicebio, Beijing, China) for 5 minutes and then thoroughly washed under running tap water for 10 minutes. Subsequently, the samples were blocked with a goat serum solution (SP KIT-B3; Fuzhou Maixin Biotech, Ltd.) for 30 minutes. The sections were then incubated with primary antibodies overnight at 4°C in a humidity-controlled chamber (REVCO; Thermo Fisher Scientific). The primary antibodies utilized included anti-amyloid-β (Aβ)1–42 antibody (rabbit monoclonal antibody, 1:100, Abcam, Cambridge, UK, Cat# ab201061, RRID: AB_2722492), anti-Tau (phospho S404) antibody (rabbit monoclonal antibody, 1:100, Abcam, Cat# ab92676, RRID: AB_10561457), anti-Iba1 antibody (rabbit recombinant antibody, 1:100, Abcam, Cat# ab178847, RRID: AB_2832244), anti-BRN3A antibody (mouse monoclonal antibody, 1:10, Sigma, St. Louis, MO, USA, Cat# MAB1585, RRID: AB_94166), and fluorescein peanut agglutinin (PNA) (1:1000, Vector Laboratories, Newark, CA, USA, Cat# FL-1071, RRID: AB_2315097). Next, the sections were washed three times with phosphate-buffered saline and soaked in wash buffer for 1 minute. Subsequently, the sections were incubated with secondary antibodies at room temperature (25–30°C) in the dark for 50 minutes. The secondary antibodies employed were Alexa Fluor 488 goat anti-rabbit IgG (1:500, Invitrogen, Carlsbad, CA, USA, Cat# A11034) and Alexa Fluor 594-conjugated donkey anti-rabbit (1:500, Invitrogen, Cat# A21207). Next, the sections were washed with PBS, and the nuclei were stained with DAPI (1:500, Sigma, Cat# D9542-5MG). Finally, the sections were rinsed three times, and the coverslips were mounted onto slides with buffered glycerol.

For Nissl staining, the paraffin-embedded brain sections were first dewaxed and rehydrated in water. Subsequently, the sections were stained with a 1% toluidine blue solution (G1436, Servicebio), dehydrated in a graded ethanol series (2 seconds in 80% ethanol, 1 minute in 95% ethanol, 1 minute in absolute ethanol I, and 1 minute in absolute ethanol II), and incubated in xylene for 20 minutes. The sections were then mounted on slides using neutral resin. For hematoxylin and eosin (H&E) staining, the sections were dewaxed and rehydrated as described above, then placed in hematoxylin staining solution (Thermo Fisher Scientific, Cat# 22-050-206) for 3 minutes, clarifier 2 solution (Thermo Fisher Scientific, Cat# 22-050-117) for 5 seconds, bluing reagent (Thermo Fisher Scientific, Cat# 6769002) for 1 minute, and eosin staining solution (Thermo Fisher Scientific, Cat# AC152880250) for 20 seconds. Finally, the sections were dehydrated as described above. All images were captured using a digital slide scanner (Olympus VS200, Olympus, Tokyo, Japan). The area of the visual field at a magnification of 200× (μm^2^) was quantified using dedicated software (OlyVIA, Olympus). Sections from the V1 and LGN visualized at 200× magnification were quantitatively evaluated using specialized software (Olympus Cell Sens Standard; Olympus). Five random 200× field of view was selected for each section, conducting cell counting. The average cell count in these five areas was calculated and normalized to the unit area (1 mm^2^) to represent the cell density in each section.

### Extracellular electrophysiological recordings

All surgical procedures were carried out in strict accordance with established guidelines. Anesthesia was induced using a gaseous anesthetic (isoflurane, 4%–5%) in closed, transparent box and maintained with a low concentration of isoflurane (isoflurane, 0.6%–0.8%) by mask. A craniotomy was performed, and a bone screw was securely affixed to the skull over the cerebellum. Tungsten electrodes (KD-SC; Kedou, Suzhou, China) were positioned as both the ground and recording electrodes on the V1 surface. To prevent desiccation and minimize brain pulsation due to respiration, the exposed cortical surfaces were covered with 2% agar before shielding to attenuate environmental interference. Animals were maintained at 37°C using warming pads, and ocular ointment (Bepanthen, Bayer AG, Germany) was regularly applied to ensure adequate moisture in their eyes throughout the procedure. Tungsten electrodes were used to record single-unit activity in anesthetized mice. As the microelectrode was slowly advanced via an electric thruster, various drifting grating patterns were presented within the animal’s visual field to stimulate the neurons. The optimal orientation, spatial frequency, temporal frequency, and receptive field size of individual V1 neurons were determined using a rigorous experimental methodology outlined in our previous study (Yang et al., 2024). Single-unit spiking activity within the frequency range of 500 to 5000 Hz was identified using an online window discriminator and precisely time-stamped with an accuracy of 1 ms using a data acquisition system (Blackrock Microsystems, Salt Lake City, Utah, USA). Principal component analysis and spike waveform characteristic analysis were employed to distinguish individual single units.

### Statistical analysis

Differences between age-matched experimental and control mice were assessed by unpaired *t*-test for histological data, whereas comparisons among different age groups were conducted by one-way analysis of variance with Tukey’s *post hoc* test. Statistical significance was defined as *P* < 0.05. Data are expressed as mean ± SD, and all analyses were performed at least three times using GraphPad Prism 9.0 software (GraphPad Software, San Diego, CA, USA, www.graphpad.com).

## Results

### Retinal degeneration in *REEP6*^–/–^ mice

**Figure 1:**
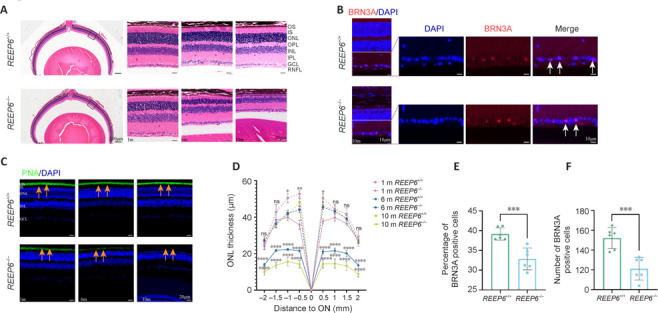
Retinal degeneration in *REEP6*^–/–^ mice was observed at 1, 6, and 10 months of age. (A) Schematic HE staining of eyeball sections from *REEP6*^–/–^ and *REEP6*^+/+^ mice was performed at each age. (B) Schematic BRN3A (red), a specific marker for RGCs, was used to stain eyeball sections from *REEP6*^–/–^ and *REEP6*^+/+^ mice at 10 months of age; white arrows show BRN3A-positive cells. (C) Schematic PNA (green), a specific marker for cones, was used to stain eyeball sections from the *REEP6*^–/–^ and *REEP6*^+/+^ groups at each age; yellow arrows indicate the layers. Scale bars are shown in figures. (D) The curve represents the thickness of the ONL at different distances from the optic nerve in both *REEP6*^–/–^ and *REEP6*^+/+^ mice at each age (comparison of ONL thickness at equivalent distances from the optic nerve between the same-aged *REEP6*^–/–^ and *REEP6*^+/+^ groups). (E, F) Comparison of the proportion and absolute number of RGCs in the ganglion cell layer between eyeball sections from the *REEP6*^–/–^ and *REEP6*^+/+^ groups at 10 months of age. Data are expressed as the mean ± SD. ****P < 0.0001. INL: Inner nuclear layer; IPL: inner plexiform layer; IS: Inner segment; m: month; ns: not significant; ONL: outer nuclear layer; OPL: outer plexiform layer; OS: outer segment; REEP6: receptor expression enhancing protein 6; RGCs: retinal ganglion cells; RNFL: retinal nerve fiber layer.

Retinal degeneration, resulting from defects in rod, cone, or retinal pigment epithelium cells, typically leads to the loss of photoreceptor cells and subsequent impairment of afferent signal transmission within the inner retina. In response, the inner retina undergoes negative synaptic plasticity, characterized initially by subtle neuronal structural modifications and subsequently by large-scale reorganization (Pfeiffer et al., 2020). To visualize overall retinal structural alterations and cell damage, retinal sections were subjected to H&E staining and immunofluorescence staining to specifically mark RGCs and cones. In the *REEP6*^+/+^ group, all layers of the retina were clearly visible, and the nuclei in the ONL were tightly arranged in regular patterns. Conversely, progressive thinning or even disappearance of the ONL, outer segment, and inner segment were seen with age increasing in the *REEP6*^–/–^ group (**Figure 1A**). These results establish that REEP6 deficiency triggers progressive retinal degeneration. To quantify the spatial and temporal dynamics of photoreceptor loss, we measured the thickness of the outer nuclear layer at various distances from the optic disc in both groups at different time points and found that, at 1 month old, there was a slight thinning only in the posterior pole of the ONL in *REEP6*^–/–^ mice (distances from the optic disc = X mm; X = –1.00 mm, *P* < 0.05; X = −0.50 mm, *P* < 0.01; X = 0.50 mm, *P* < 0.05; **Figure 1D**). At 6 and 10 months, significant thinning of both the central and peripheral positions of the outer nuclear layer was observed (all measurement positions, *P* < 0.0001; **Figure 1D**). This demonstrates that photoreceptor degeneration in *REEP6*^–/–^ mice initiates in the mid-peripheral retina and rapidly progresses to involve the entire ONL. By 10 months, only one or two retinal layers exhibited disordered nuclei, while both the outer segment and inner segment had disappeared. Additionally, there appeared to be an unclear boundary between the ONL, INL, and OPL.

Next, we performed PNA immunofluorescence staining to assess cone retention at various time points, because PNA specifically binds to cones in the retina. Compared with *REEP6*^+/+^ mice, *REEP6*^–/–^ mice exhibited a slight reduction in cone numbers at 1 month of age, followed by significant loss of cones at 6 months and near disappearance of cones at 10 months (**Figure 1C**). These data indicate that cone photoreceptors are not spared and undergo progressive degeneration following the initial loss of photoreceptors (primarily rods) in the ONL. Furthermore, BRN3A immunofluorescence staining of retinal ganglion cells (RGCs) showed that, while there were no significant differences in the number of BRN3A-positive RGCs between the *REEP6*^–/–^ and *REEP6*^+/+^ groups at 1 and 6 months of age, there was a statistically significant decrease in the number of BRN3A-positive RGCs at 10 months of age (**Figure 1B**) in *REEP6*^–/–^ mice compared with *REEP6*^+/+^ mice (*P* < 0.001, **Figure 1E** and **F**). This observation suggests that RGC loss is a late event in *REEP6*^–/–^ mice-induced retinal degeneration, occurring only after advanced photoreceptor loss and laminar disorganization, consistent with progression to end-stage disease and remodeling of the inner retinal layer.

### Microglia and phosphorylated Tau participate in retinal degeneration in *REEP6*^–/–^ mice

**Figure 2:**
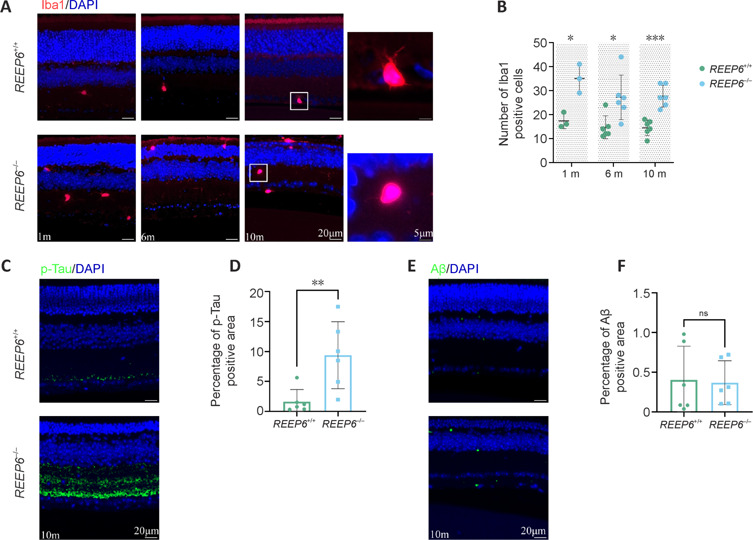
Microglia and p-Tau contribute to retinal degeneration in *REEP6*^–/–^ mice. (A) Schematic diagram illustrating the specific labeling of microglia (Iba1, shown in red) in eyeball sections of *REEP6*^–/–^ and *REEP6*^+/+^ mice in different age groups. Resting and activated microglia are enlarged for clarity (indicated by white arrows). (B) The total number of Iba1-positive cells was compared between *REEP6*^–/–^ and *REEP6*^+/+^ mice in each age group. (C) Schematic diagram depicting the staining of p-Tau (shown in green) in eyeball sections of 10-month-old *REEP6*^–/–^ and *REEP6*^+/+^ mice. (D) The area ratio of p-Tau deposition was compared between 10-month-old *REEP6*^–/–^ and *REEP6*^+/+^ mice. (E) Schematic diagram showing the staining of Aβ (shown in green) in the same model mice. (F) The area ratio of Aβ deposition was compared between 10-month-old *REEP6*^–/–^ and *REEP6*^+/+^ mice. Data are expressed as the mean ± SD. *P < 0.05, **P < 0.01, ***P < 0.001. Scale bars are provided within the figures. Aβ: Amyloid-β; m: month(s); ns: not significant; p-Tau: phosphorylated tau.

Microglia, which serve as the primary immune surveillance and clearance cells in the brain (Madsen et al., 2024), were examined to elucidate their role in retinal degeneration. Retinal sections were subjected to microglia-specific Iba1 immunofluorescence staining. Our results demonstrated that microglia in *REEP6*^+/+^ mice predominantly localized to the inner layer of the retina, whereas in *REEP6*^–/–^ mice, they were distributed across all layers of the retina and primarily exhibited an activated phenotype (**Figure 2A**). Statistical analysis revealed a significant increase in the number of microglia within the retinas of *REEP6*^–/–^ mice at 1, 6, and 10 months compared with *REEP6*^+/+^ mice (number of Iba1-positive cells: 1 month, *REEP6*^–/–^ *vs.* *REEP6*^+/+^, *P* < 0.05; 6 months, *REEP6*^–/–^ *vs.* *REEP6*^+/+^, *P* < 0.05; 10 months, *REEP6*^–/–^ *vs. REEP6*^+/+^, *P* < 0.001; **Figure 2B**). However, no significant differences were observed in the number of microglia across different age groups of *REEP6*^–/–^ mice (*P* > 0.05 for all comparisons). These findings demonstrate that *REEP6* double knockout in mice triggers an early, robust, and sustained microglial response characterized by increased microglia cell numbers, widespread migration into outer retinal layers, and morphological activation.

Accumulation of Aβ and p-Tau is a hallmark of neurodegenerative diseases. To explore their deposition in retinal degeneration, retinal sections were subjected to Aβ and p-Tau immunofluorescence staining. The analysis revealed no significant differences in p-Tau accumulation in 6-month-old *REEP6*^–/–^ and *REEP6*^+/+^mice (*P* > 0.05, data not shown). In contrast, a significant increase in p-Tau was detected within the inner plexiform layer (IPL) region in 10-month-old *REEP6*^–/–^ mice compared with their age-matched *REEP6*^+/+^ counterparts (percentage of p-Tau positive area (%): *REEP6*^–/–^ *vs*. *REEP6*^+/+^, *P* < 0.01; **Figure 2C** and **D**). Furthermore, the p-Tau positive area of 10-month-old *REEP6*^–/–^ mice was significantly increased compared with that of 10-month-old *REEP6*^+/+^ mice (data not shown). This indicates that p-Tau accumulation is a late-occurring pathological event in *REEP6*^–/–^-induced retinal degeneration, manifesting only in the advanced stage after photoreceptor loss and microglial activation. Immunofluorescence staining for Aβ demonstrated minimal amounts of Aβ monomers in the retinas of both *REEP6*^–/–^ and *REEP6*^+/+^ mice at 10 months of age, with no detectable Aβ plaques and no statistically significant differences between the two groups at this time point (**Figure 2E** and **F**; similar results in 1 and 6 months groups, data not shown). These results demonstrate that, unlike p-Tau accumulation, significant Aβ accumulation and plaque formation is not a characteristic feature of retinal degeneration in the *REEP6*^–/–^ mouse model.

### Nissl staining and ionized calcium-binding adapter molecule 1, phosphorylated Tau, amyloid-β staining of the lateral geniculate nucleus and primary visual cortex

**Figure 3:**
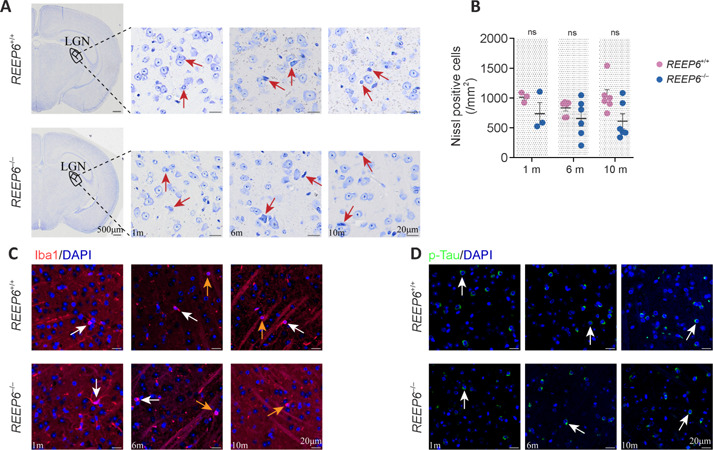
Representative images of Nissl staining and Iba1, p-Tau, and Aβ immunostaining of LGN sections from *REEP6*^–/–^ mice. (A) Nissl staining of LGN sections from *REEP6*^–/–^ and *REEP6*^+/+^ mice in each age group; neurons containing Nissl bodies are indicated by red arrows. (B) Comparison of the density of cells containing Nissl bodies in LGN sections from *REEP6*^–/–^ and *REEP6*^+/+^ mice in each age group. (C) Iba1-immunoreactive microglia (red) in LGN sections from both *REEP6*^–/–^ and *REEP6*^+/+^ mice in each age group, with resting microglia indicated by white arrows and activated microglia indicated by yellow arrows. (D) p-Tau immunoreactive cells (green) in LGN sections from both *REEP6*^–/–^ and *REEP6*^+/+^ mice in each age group are shown by white arrows. Data are expressed as the mean ± SD. Scale bars are provided within the figure. LGN: Lateral geniculate nucleus; m: month; ns: not significant; p-Tau: phosphorylated tau; REEP6: receptor expression enhancing protein 6.

Nissl bodies, which are basophilic cytoplasmic structures found in neurons, display a wide range of morphologies, sizes, and quantities across different neuronal cell types. Nissl staining facilitates visualization of nerve cell architecture and assists in evaluating neuronal damage. Neurons with a greater abundance of Nissl bodies typically exhibit enhanced protein synthesis and increased neural activity (Magnain et al., 2014). Nissl staining was used to assess the protective damage on neurons. Moreover, p-Tau, Aβ and microglia-specific immunofluorescence staining was used to assess the hallmark involving in neuronal degeneration in brain. No significant differences were observed in the number of Nissl-containing neurons in the LGN between the *REEP6*^–/–^ and *REEP6*^+/+^ groups at all age tested, and the neurons exhibited comparable cell morphology (**Figure 3A** and **B**). These findings demonstrate that neuronal architecture and density were preserved in the LGN despite advanced retinal degeneration. To explore potential biomarkers associated with LGN neurodegeneration in *REEP6*^–/–^ mice, we performed Iba1 immunofluorescence staining a found minimal numbers of microglia in the LGN of both *REEP6*^–/–^ and *REEP6*^+/+^ mice at different ages, with no significant pairwise differences; additionally, a few of activated microglial cells were detected both *REEP6*^–/–^ and *REEP6*^+/+^ mice (**Figure 3C**). No Aβ was detected by immunofluorescence staining (data not shown), and p-Tau staining revealed no significant changes in p-Tau levels between the two groups (**Figure 3D**). No microglial activity or pathological protein accumulation was detected in the LGN of *REEP6*^–/–^ mice.

**Figure 4:**
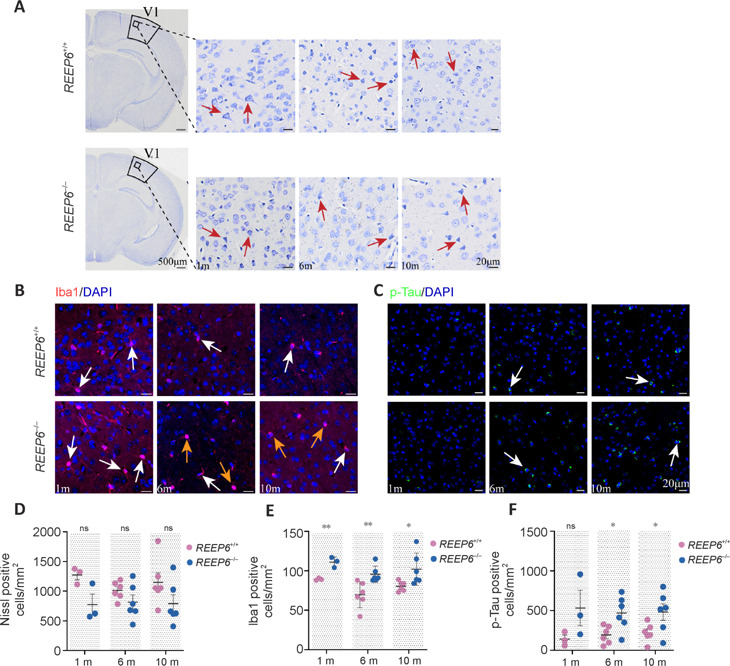
The number and activation of microglia in the primary visual cortex (V1) of *REEP6*^–/–^ mice exhibited an increase, accompanied by an elevation in intracellular p-Tau deposition. (A, D) No significant difference in Nissl staining of V1 sections was detected between *REEP6*^–/–^ and *REEP6*^+/+^ mice across different age groups, with red arrows indicating neurons containing Nissl bodies. (B, E) Iba1-immunoreactive microglia (white arrows), specifically labeling microglia, in V1 sections of *REEP6*^–/–^ and *REEP6*^+/+^ mice at various ages. (C, F) p-Tau Iba1-immunoreactive cells (white arrows) in V1 sections from both *REEP6*^–/–^ and *REEP6*^+/+^ mice at different ages. Scale bars are provided within figures. Data are expressed as the mean ± SD. *P < 0.05, **P < 0.01. m: Month; ns: not significant; p-Tau: phosphorylated Tau; REEP6: receptor expression enhancing protein 6; V1: primary visual cortex.

To investigate the impact of retinal degeneration on V1, we performed Nissl staining to assess changes in neuron quantity and activity. Additionally, Iba1, Aβ, and p-Tau immunofluorescence staining were employed to evaluate neurodegeneration in V1. No significant differences were observed in the number of Nissl-positive neurons in V1 between the *REEP6*^–/–^ and *REEP6*^+/+^ groups at all time points tested, and the neurons exhibited comparable cell morphology in both groups (**Figure 4A** and **D**). These findings indicate that neuronal numbers and structural integrity were maintained in V1 throughout retinal degeneration. Immunofluorescence staining for Iba1 revealed increased microglia abundance, denser microglia distribution, and larger microglia cell bodies in V1 in *REEP6*^–/–^ mice compared with *REEP6*^+/+^ mice at different time points (**Figure 4B**). Statistical analysis showed significant differences in microglial abundance between the two groups at all time points tested (1 month, *REEP6*^–/–^ *vs.* *REEP6*^+/+^, *P* < 0.01; 6 months, *REEP6*^–/–^ *vs. REEP6*^+/+^, *P* < 0.01; 10 months, *REEP6*^–/–^ *vs*. *REEP6*^+/+^, *P* < 0.05; **Figure 4E**). This demonstrates early, sustained microglial activation in V1 that parallels retinal degeneration. Furthermore, Aβ and p-Tau immunofluorescence staining showed a higher density of p-Tau-positive cells in V1 in *REEP6*^–/–^ mice compared with *REEP6*^+/+^ mice at 6 and 10 months (density of p-Tau-positive cells: 6 months, *REEP6*^–/–^ *vs. REEP6*^+/+^, *P* < 0.05; 10 months, *REEP6*^–/–^ *vs*. *REEP6*^+/+^, *P* < 0.05); however, this difference was not observed at 1 month (**Figure 4C** and **F**). There were no statistically significant differences in Aβ optical density value between the V1 regions of *REEP6*^–/–^ and *REEP6*^+/+^ mice at any time point (*P* > 0.05, data not shown). Thus, p-Tau accumulation in V1 occurs late in retinal degeneration, without concomitant Aβ accumulation, suggesting distinct mechanisms of cortical proteinopathy.

### Increase in spontaneous activity and decreased visually-induced spiking in the primary visual cortex

**Figure 5:**
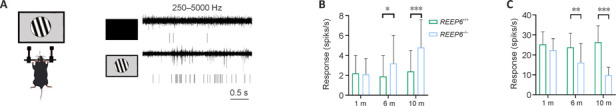
Spontaneous and visually evoked neural activity observed at different time points. (A) Under light anesthesia, single-cell recordings were performed to quantify spontaneous activity in the absence of any external stimulus and measure the maximum response elicited by the receptive field corresponding to an individual neuron. (B) Spontaneous activity of *REEP6*^–/–^ mice at different time points. (C) Grating-induced response of neurons in the primary visual cortex under classical receptive field stimulus in *REEP6*^–/–^ mice. Data are expressed as the mean ± SD. *P < 0.05, **P < 0.01, ***P < 0.001. m: Month(s); REEP6: receptor expression enhancing protein 6.

During RP progression, the gradual degeneration of rods leads to retinal degeneration, resulting in the remodeling of entire visual pathways and ultimately causing significant alterations in neural activity within the CNS (Wang et al., 2016). Thus, we investigated spontaneous neuronal firing and grating-induced spiking in *REEP6*^–/–^ and *REEP6*^+/+^mice at different ages. The mice were lightly anesthetized, and the neural activity of individual neurons was quantified in different layers of the V1 region through extracellular recordings. During tests for spontaneous activity, neuron responses were recorded in a dark room without any stimuli present. The optimal stimulus conditions were initially determined for each neuron using visually induced recordings. The maximum response was determined by calculating the firing rate of a single neuron corresponding to its receptive field (**Figure 5A**). Ten to sixteen distributed across the II/III, IV, and VI/VII layers were recorded for each mouse. No significant difference in spontaneous activity was observed between the experimental group and the control group at 1 month of age (*REEP6*^–/–^ *vs. REEP6*^+/+^, *P* > 0.05; **Figure 5B**). However, at 6 months and 10 months of age, the *REEP6*^–/–^ group exhibited significantly higher levels of spontaneous activity compared with the *REEP6*^+/+^ group (6 months: *P* < 0.05; 10 months, *P* < 0.001). Furthermore, there was a significant age-dependent increase in spontaneous activity in the *REEP6*^–/–^ group compared with the *REEP6*^+/+^ group (*P* < 0.05). These results demonstrate progressive development of cortical hyperexcitability coinciding with advancing retinal degeneration. Additionally, there was no statistically significant difference in the maximum grating-induced response between the groups in 1-month-old mice (*P* > 0.05; **Figure 5C**). Nevertheless, in 6-month-old and 10-month-old mice, the *REEP6*^–/–^ group demonstrated a significantly lower response compared with the *REEP6*^+/+^ group (6 months: *P* < 0.01; 10 months: *P* < 0.001). The maximum grating-induced response in *REEP6*^–/–^ mice was observed at 3 months and showed a significant difference compared with that of *REEP6*^+/+^ mice; this response gradually declined as eye degeneration progressed (*P* < 0.01). This findings suggest severe, age-dependent impairment in visual processing capacity, despite intact early-stage function. The overall spiking activity in V1 observed in our *REEP6*^–/–^ mice aligns with findings from previous models reporting a predominant presence of spontaneous activity and a diminished response to visual stimuli. Taken together, our findings demonstrate a pathological shift in V1 activity patterns over time: baseline spontaneous firing progressively increased, while visually-driven responses simultaneously deteriorated in *REEP6*^–/–^ mice from 6 months onward. This reciprocal pattern of heightened spontaneous activity coupled with diminished sensory processing mirrors the characteristic cortical remodeling that takes place in models of advanced retinal degeneration.

## Discussion

Most neurodegenerative diseases have characteristic abnormal protein aggregation, that is closely related to changes in multiple molecular pathways and pathological features. The special structural and functional connections between the eyes and the brain make them share many similarities in the occurrence of diseases. These diseases, such as AD, Parkinson’s disease, and other brain disorders, share many similarities in molecular pathological mechanisms with retinal diseases such as AMD and glaucoma (Sivak, 2013). Based on this, we explore the role of common neurodegenerative disease pathogenesis in the neurodegeneration and visual pathway damage of RP. Studies have found that both the retina and the V1 area show an increase in the number of microglia and intensified activation, accompanied by an increase in p-Tau deposition, indicating that they jointly participate in the neurodegenerative pathological changes of the RP visual pathway. In terms of function, spontaneous neural activity in the V1 area is enhanced, but visually-driven responses is weakened. It is suggested that tau and microglia play a role in the retinal neurodegeneration linked to RP, even in the V1, where a comparable neurodegeneration occurs along with functional impairment of the entire visual pathway.

REEP6 is specifically expressed in rod cells, where it serves as a crucial mediator of vesicle transport. Grid protein–encapsulated vesicles carrying REEP6 are selectively transported to membrane sites with Syntaxin3, resulting in impaired function and eventual demise of the photoreceptors when REEP6 is absent (Veleri et al., 2017). A study investigating the association between *REEP6* and RP found slight thinning of the ONL at P20 in *REEP6*^–/–^ mice that progressively worsened with age. By 2 months, *REEP6*^–/–^ mice exhibited evident retinal degeneration, significantly thinner ONL, and a reduced number of nuclear layers compared with both *REEP6*^+/+^ and *REEP6*^+/−^ mice. At 4 months, only 40% of the ONL remained intact. Transmission electron microscopy analysis demonstrated a substantial reduction in the number of photoreceptors by 6 months, accompanied by structural disorganization characterized by shortened and fragmented outer segments. In contrast, the retinas of *REEP6*^+/−^ mice appeared structurally normal (Agrawal et al., 2017). The results from our study align with this description of disease progression, suggesting that *REEP6*^–/–^-related RP progresses rapidly. Despite the short life cycle of mice, the terminal stage is also easily observable.

The retinal degeneration and remodeling associated with hereditary retinal diseases (such as RP), genetic and environmental factors (such as AMD), as well as other ocular/retinal injuries (including trauma and retinal detachment) encompass a range of pathological changes at the molecular, cellular, and tissue levels. Retinal remodeling is akin to the negative plasticity that occurs in CNS disorders such as trauma and epilepsy, posing a major obstacle to all types of rescue strategies. Retinal remodeling involves three levels of reorganization in the sensory layer of the retina: neuronal cell death, cellular migration, and rewiring. Upon stimulation, photoreceptor cells undergo transient neurite sprouting and extension into the inner retina towards the IPL layer before retracting into neurites and residual synaptic anterior terminals prior to cell death (Marc et al., 2003). In RP models, pathogenesis initiates with the death of rod cells and subsequent cone cell degeneration, presumably because rod cells provide survival factors for cone cells (Mohand-Said et al., 1998). Moreover, the demise of rod cells triggers the activation and recruitment of resident retinal microglial cells to the outer nuclear layer, resulting in widespread cellular death (Díaz-Lezama et al., 2023). The pivotal event triggering long-term remodeling of nerve cells and glia in residual neuropathic retina is the loss of cone photoreceptors; as such, this is a main focus of therapeutic approaches. RGCs are insensitive to photoreceptor stimulation and death in the early stages of RP, only exhibiting changes in number and structure at later stages. Because few studies have been carried out in aging animals, many models assume that RGCs are unaffected by RP. Retinal remodeling has been observed in various models, such as the TgP347L rabbit model and the P23H pig model (Jones et al., 2011; Ross et al., 2012). However, detailed information regarding retinal remodeling in *REEP6*^–/–^ animals (a recently developed experimental model) remains scarce. In this study we found that the retinas of 6-month-old *REEP6*^–/–^ mice are in stage 2 of retinal remodeling, characterized by a rapid decrease in cone cells. By 10 months old, cones are nearly absent, the number of RGCs is decreased, and the remodeling process has entered an irreversible long-term stage, with more severe glial occlusion. Our findings support previous hypotheses regarding the reduction in RGCs in RP. Thus, *REEP6*^–/–^ mice could be used as models for studying the efficacy of potential treatments. Furthermore, our findings offer a reference point for investigating the histopathological and structural characteristics of the retina and its connections to the LGN to V1 at different stages in *REEP6*^–/–^ mice.

Microglia are the predominant immune cells in the retina. Under pathological conditions, such as retinal degeneration, microglia are activated by various stress-related and neurodegenerative triggers (Tolentino et al., 2023; Qian et al., 2025). The transition from a dendritic to an amoeboid morphology serves as a pivotal indicator of microglial activation, as the amoeboid form enables them to proliferate and migrate, ultimately resulting in microglial infiltration into and accumulation in the ONL (Zou et al., 2019). Activated microglia possess robust phagocytic capabilities and play a crucial role in synaptic pruning during CNS development. Similarly, in a classic RP model, microglia were found to participate in the complement-dependent clearance of synaptic components and delay the formation of ectopic neurons, as well as visual function deterioration, during retinal degeneration (He et al., 2019). Additionally, microglia can induce neuroinflammatory activation and assume a neurotoxic phenotype, contributing to AMD progression to its advanced stage (Wu et al., 2021; Dhodapkar et al., 2022). *CX3CR1*^–/–^ mice exhibited significantly increased microglial infiltration, inflammatory cytokine secretion, and phagocytic activity in the outer nuclear layer, consequently accelerating photoreceptor degeneration and death (Zabel et al., 2016). In our study, we observed microglial activation, increased microglia numbers, and microglia migration towards the ONL in the retinas of 1-month-old *REEP6*^–/–^ mice. These changes may be associated with early-stage photoreceptor degeneration and cell death. Microglia play a crucial role in the abnormal extension and retraction of photoreceptor synapses, as well as bipolar and horizontal cell synapse retraction, during retinal remodeling.

Amyloid and Tau accumulation are the main mechanisms thought to be involved in AD pathology. Microglia may play a preventive role, as they promote Aβ clearance, inhibit Tau hyperphosphorylation, and produce neurotrophic factors, thereby delaying the onset of AD symptoms (Zhang et al., 2013; Merlo et al., 2018; Delizannis et al., 2021; Wang et al., 2024). In this study, we found that p-Tau deposition in 10-month-old *REEP6*^–/–^ mice was significantly increased compared with the control group, while Aβ accumulation did not differ significantly between the groups throughout the experiment. Given that the eyes are an extension of the CNS, the pathology of retinal degeneration seems to involve a mechanism similar to that of AD, i.e., microglia phagocytose and disseminate Tau (Hanslik and Ulland, 2020). Proinflammatory cytokines produced by active microglia, such as IL-1 and IL-6, cause Tau phosphorylation, but are insufficient to promote the formation of neurofibrillary tangles in the retina. In summary, both defects in Tau clearance and neuroinflammatory mechanisms that promote Tau aggregation and proliferation contribute to microglia-induced Tau-related disease (Guo et al., 2022). Our results are consistent with an earlier study suggesting that abnormal p-Tau accumulation may disrupt cone function (Aboelnour et al., 2017). In addition, the observed increase in p-Tau levels in the ganglion cell layer and IPL in retinal degeneration model (Chiasseu et al., 2016; Zhu et al., 2018), as well as in horizontal cells and the IPL in glaucoma cases (Gupta et al., 2008), emphasizes the relevance of pathological Tau to RGC degeneration. The cone and RGC degeneration that we observed in the retina support this view to a certain extent.

The possible interconnection between the eyes and the CNS has been a topic of discussion for many years. Some studies have shown that, on one hand, the neurodegeneration and proteinopathy that occur during retinal degeneration are similar to those seen in CNS neurodegenerative diseases, while on the other hand, CNS diseases, which have high incidence and mortality rates worldwide, involve specific changes at the eye level. AD and other types of dementia all involved the formation of protein aggregates, a common pathological feature of neurodegenerative diseases (Xiang et al., 2023). AD is the CNS disease that is most often mentioned in relation to RD, especially AMD, because both are strongly associated with age, and some studies have even found Aβ deposits in the eyes of patients with AMD (Dentchev et al., 2003). In RD, protein aggregates are thought to induce the death of photoreceptor cells (Illing et al., 2002; Saliba et al., 2002).

In this study, we asked whether similar pathogenic factors affect LGN and V1 neurons at different stages of retinal degeneration in *REEP6*^–/–^ mice. The number of microglia in V1 was higher in 1, 6, and 10-month-old *REEP6*^–/–^ mice than in *REEP6*^+/+^ mice at the same ages, but no such difference was observed in LGN. This may be related to the significant decrease in dendritic spine density in V1 and the change in the distribution of dendritic spines along the axis of cone neurons in RP (Martinez-Galan et al., 2022). In addition, One study showed that visual deprivation in deaf cats resulted in a significant reduction in the number of dendritic spines on the thalamic cortex (Antonini and Stryker, 1993). This process requires activation of small glia cells, which help prune neural cell synapses. Furthermore, we found that the number of cells containing p-Tau in the V1 region of 6-month-old and 10-month-old *REEP6*^–/–^ mice was increased compared with age-matched *REEP6*^+/+^ mice, but there was no increase in Aβ deposition. The synergistic role of microglia cells and Tau in neurodegenerative processes is well-established, and both play an important role in AD pathology; in particular, microglia are believed to be involved in cognitive functional decline (Udeochu et al., 2023; Bedolla et al., 2024).

The LGN is a key element of the visual pathway, but was not affected in *REEP6*^–/–^ mice according to several of the indicators assessed in this experiment. This may be because different cells have different sensitivities to visual impairment. Previous studies have suggested that the nerve cells in layers III and V of V1 are sensitive to changes in visual experience, and that the number of nerve cell dendritic spines changes during visual deprivation (Heumann and Rabinowicz, 1982; Macharadze et al., 2019). Because RGC degeneration was not been observed in the early stage of the disease, and no nerve cell degeneration in the LGN was observed throughout the course of the disease, the pathological changes that we detected in V1 likely cannot be explained by the transsynaptic degeneration as occurs in glaucoma (Sacca et al., 2020; Chan et al., 2021). Our findings suggest that, when the visual activity of V1 nerve cells is abnormal, there will be an increase in microglia numbers and microglial activation, as well as an increase in p-Tau, all of which produce degenerative changes similar to those seen in degenerative diseases of the CNS.

Electrophysiological assessment of animal models of retinal degeneration has shown that progression of this condition not only affects the retina itself, but also V1, potentially leading to an imbalance in the inhibitory-excitatory system (Stasheff et al., 2011; Wang et al., 2016). This imbalance manifests as faster and more regular spontaneous activity, suggesting a reduced ability of the cortical visual system in RD models to generate new neural activity patterns. These patterns are related to both instantaneous responses to stimuli and persistent circuit changes dependent on neural plasticity (Wang et al., 2016). V1 neurons in retinal degeneration models demonstrates an increased firing rate, indicating potential alterations along the entire visual pathway. In this study, we explored the spontaneous activity of V1 neurons and found that, there was significantly higher levels of spontaneous activity along with retinal degeneration; indicating that the ability of V1 to generate new neural activity patterns has declined in the RP model. In this study, we found that V1 neurons in *REEP6*^–/–^ mice showed significantly weakened visual-evoked activity and impaired visual perception ability in the middle and late stages of RD. This may be related to degenerative damage to the entire visual pathway. However, structural damage to the retina itself reduces the ability to receive visual stimulation, which also affects the intensity of neural electrical activity measured in the V1 region. Therefore, further studies are needed to evaluate the functional status of V1 neurons.

There were some limitations to this study. Firstly, as the LGN is an important relay station for the transmission of visual signals to the brain, the absence of observed changes to LGN histology in the RP model mice was unexpected. This may be because the LGN is highly tolerant to visual impairment, or because of inadequate experimental methods. Future studies should use improved methods for LGN electrophysiological recordings. Additionally, it is challenging to ascertain whether the defects observed in the cortex were caused by inadequate visual input from the retina or by local degeneration of cortical neurons. In the future, we will consider using electrode or optogenetic stimulation of the LGN and recording the V1 response to address both possibilities and carefully analyze the origin of changes in spontaneous activity. Two-photon imaging has unique advantages for evaluating this type of activity and could be used in future studies (Somarowthu et al., 2021).

In conclusion, the RGC morphology findings from the present study of *REEP6*^–/–^ mice support the hypothesis that RP leads to a reduction in the number of retinal RGCs. Moreover, we observed hallmark pathologies of brain degeneration, such as p-Tau accumulation and microglia infiltration, after the gradual death of rod cells in the *REEP6*^–/–^ mouse model. The presence of both heightened microglial activation and increased microglial numbers in both the retina and V1, along with increased p-Tau deposition, suggests their joint involvement in the pathological alterations associated with neurodegeneration of the RP visual pathway. Additionally, we observed enhanced spontaneous activity and diminished visually-induced activity, which further indicate compromised neuronal function within the V1 region, implying that impairment of RP function throughout the visual pathway may encompass V1. Notably, there were certain limitations to this study in terms of studying RP-related neurodegeneration and aberrant electrical activity in the visual pathway, and more research is required to clarify these points in the future. Currently, there are no effective treatments available to prevent RP development and progression or restore vision. However, given the increasing life expectancy, the effects of age-related visual impairment on quality of life merit consideration. Our findings provide new insight into treatment timing and could help development treatments for RP and even RD by clarifying the pathological and structural effects of RP on the retina, LGN, and V1.

## Data Availability

*No additional data are available.*
